# Interpreting salivary cortisol thresholds in ectopic ACTH syndrome

**DOI:** 10.1530/EC-26-0399

**Published:** 2026-07-15

**Authors:** Omer Denizhan Tatar

**Affiliations:** Department of Endocrinology and Metabolism, Necmettin Erbakan University Faculty of Medicine, Konya, Türkiye

**Keywords:** ectopic ACTH syndrome, Cushing’s syndrome, late-night salivary cortisol, survival, assay variability

## Abstract

This Perspective comments on a recent national cohort study of ectopic ACTH syndrome that identified late-night salivary cortisol (LNSC) ≥ 130 nmol/L as an independent predictor of mortality. The finding is clinically important because it supports the prognostic relevance of biochemical severity. However, its numerical interpretation should remain method-specific. LNSC is sensitive to sampling conditions and analytical platform, and absolute values are not necessarily interchangeable across immunoassays or between immunoassays and liquid chromatography–tandem mass spectrometry. In addition, dichotomizing a continuous biomarker can reduce information and overstate apparent separation at a data-derived cut point. Building on the original authors’ own assay caveat, the threshold is best viewed as a Roche ECLIA-context signal of severe hypercortisolism, pending prospective multicenter validation using continuous biomarker modeling, assay harmonization, and subgroup analyses by tumor context and treatment era.

## Main text

Belaya *et al.* provided one of the most informative recent datasets in ectopic ACTH syndrome (EAS), a disorder in which prognosis is shaped by both tumor biology and the systemic consequences of cortisol excess. In their national retrospective cohort of 173 patients, 50 deaths occurred during follow-up, and the reported 3- and 5-year survival rates were 77 and 70%, respectively (1). The multivariable Cox model identified age at diagnosis ≥ 51 years, distant metastases, active hypercortisolism, and late-night salivary cortisol (LNSC) ≥ 130 nmol/L as factors associated with mortality (1). That message is clinically valuable: biochemical severity is not a secondary detail in EAS, but part of the prognostic phenotype. The original report also explicitly cautioned that the threshold is meaningful only within the Roche electrochemiluminescence immunoassay (ECLIA) context. This Perspective therefore builds on that caveat, rather than presenting assay dependence as an overlooked flaw. The clinical question is how such a threshold should be translated beyond the setting in which it was derived.

The first reason is analytical. LNSC is an established test in the diagnostic evaluation of Cushing’s syndrome, but its interpretation depends on preanalytical conditions, sampling time, sleep pattern, contamination, comorbid states, and analytical platform (2). In the study by Belaya *et al.*, the prognostic threshold was generated within the measurement environment of the authors’ center and was linked to the Roche ECLIA platform (1); the authors also stated directly that this threshold was meaningful only for that Roche ECLIA context. This acknowledgment is important for implementation because salivary cortisol and cortisone concentrations, reference intervals, and diagnostic performance vary according to the assay used; values obtained by different immunoassays or by liquid chromatography–tandem mass spectrometry should not be assumed to be numerically interchangeable (3). A single value can therefore appear more definitive than the underlying biology and calibration justify. For clinical translation, the more defensible conclusion is that markedly elevated LNSC identifies a high-risk biochemical phenotype within a defined analytical context, but not that 130 nmol/L is a general boundary applicable across laboratories.

The second reason is statistical. Cortisol excess behaves biologically as a continuum, whereas a binary threshold forces patients on either side of one cut point into distinct groups. Dichotomizing continuous predictors can reduce power, discard prognostic information, and create an overconfident impression of risk separation, especially when the cut point is derived from the same dataset in which its prognostic value is evaluated (4). This does not invalidate the authors’ observation. Rather, it changes how the result should be used. The reported threshold is a useful signal that severe biochemical activity is associated with worse survival, but it should be considered hypothesis-generating until it is tested with prespecified modeling in independent cohorts. Flexible continuous modeling, spline-based approaches, or clinically prespecified categories would help determine whether risk rises gradually, accelerates above a certain range, or differs by tumor subtype.

The third reason is clinical heterogeneity. The cohort covers 1990–2024, a long period during which neuroendocrine neoplasm classification, cross-sectional and functional imaging, pathology reporting, cortisol-lowering therapy, perioperative care, and adrenal-directed strategies changed substantially (1, 5, 6). EAS is also not a single biological entity. Recent pooled evidence suggests that survival differs markedly by tumor origin, with reported 5-year survival probabilities of 81% for pulmonary neuroendocrine neoplasms, 66% for occult disease, 50% for thymic tumors, and 40% for pancreatic tumors (5). Multicenter data similarly indicate that tumor type, grading, distant metastases, severity of hypercortisolism, hypokalemia, and diabetes mellitus all contribute to outcome (7). A pooled model is useful for identifying broad prognostic signals, but it cannot fully separate endocrine severity from tumor biology, metastatic burden, diagnostic era, and treatment opportunity. That limitation is especially relevant when a biochemical cutoff is proposed for clinical use.

A related issue is treatment interpretation. The study appropriately emphasizes the importance of controlling hypercortisolism, but retrospective survival associations should not be converted too readily into treatment effects. Persistent hypercortisolism, tumor resection, and bilateral adrenalectomy are influenced by operability, frailty, infection, disease tempo, local expertise, access to care, and the time a patient must survive before receiving an intervention (1, 7). In observational analyses, these features raise the possibility of confounding by indication and time-related bias, including immortal time bias, if follow-up, treatment assignment, and eligibility are not aligned in a target trial framework (8). The practical implication is not therapeutic nihilism. On the contrary, uncontrolled cortisol excess in EAS requires urgent attention. The caution is that survival models from retrospective cohorts can support the need for rapid hormonal control, but they cannot by themselves identify the treatment strategy most likely to improve survival.

The lasting value of Belaya *et al.*’s study is therefore substantial but should be framed precisely. It shows that biochemical severity, including very high LNSC, carries prognostic information in EAS (1). Its most cautious translation is that very high LNSC, when measured in a defined analytical context, can contribute to risk stratification, but fixed numerical thresholds should not be exported across laboratories without method-specific validation. Future studies would be strengthened by multicenter assay harmonization, reporting of method-specific reference intervals, prespecified continuous biomarker modeling, sensitivity analyses across tumor subgroups, and explicit handling of treatment era effects. Such work would preserve the central clinical insight of the study while defining how far a specific LNSC threshold can travel beyond the setting in which it was derived.

## Declaration of interest

The author declares that there is no conflict of interest that could be perceived as prejudicing the impartiality of this article.

## Funding

This research did not receive any specific grant from any funding agency in the public, commercial, or not-for-profit sector.

## Author contribution statement

ODT conceived the article, reviewed the relevant literature, drafted the manuscript, critically revised it for intellectual content, approved the final version, and accepts responsibility for the work.

## Ethics statement

Ethical approval and informed consent were not required because this Perspective contains no original human participant data, patient-level data, clinical images, or intervention.
